# YAP-dependent Wnt5a induction in hypertrophic adipocytes restrains adiposity

**DOI:** 10.1038/s41419-022-04847-0

**Published:** 2022-04-27

**Authors:** Gwan-Jun Lee, Youn Ju Kim, Bongju Park, Sujin Yim, Chansang Park, Hyunsoo Roh, Yunwon Moon, Je Kyung Seong, Hyunsung Park

**Affiliations:** 1 Department of Life Science, University of Seoul, Seoul, 02504 Republic of Korea; 2 Laboratory of Developmental Biology and Genomics, BK21 Program for Veterinary Science, College of Veterinary Medicine, Seoul National University, Seoul, Republic of Korea; 3 Korea Mouse Phenotyping Center (KMPC), Seoul National University, Seoul, 08826 Republic of Korea; 4 Interdisciplinary Program for Bioinformatics, Program for Cancer Biology and BIO-MAX/N-Bio Institute, Seoul National University, Seoul, 08826 Republic of Korea; 5 Department of Applied Chemistry, University of Seoul, Seoul, 02504 Republic of Korea; 6 Present Address: Cellid, Inc., Seoul, 08826 Republic of Korea

**Keywords:** Cell signalling, Metabolism

## Abstract

Wnt5a, a prototypic non-canonical Wnt, is an inflammatory factor elevated in the sera of obese humans and mice. In the present study, fat-specific knockout of Wnt5a (Wnt5a-FKO) prevented HFD-induced increases in serum Wnt5a levels in male C57BL/6 J mice, which suggested adipocytes are primarily responsible for obesity-induced increases in Wnt5a levels. Mouse subcutaneous white adipose tissues (WATs) more sensitively responded to HFD, in terms of cell size increases and Wnt5a levels than epididymal WATs. Furthermore, adipocyte sizes were positively correlated with Wnt5a levels in vitro and in vivo. In hypertrophic adipocytes, enlarged lipid droplets increased cell stiffness and rearranged the f-actin stress fibers from the cytoplasm to the cortical region. The activities of YAP (Yes-associated protein) and TAZ (transcriptional co-activator with PDZ-binding motif) increased in response to these mechanical changes in hypertrophic adipocytes, and inhibition or knock-down of YAP and TAZ reduced Wnt5a expression. ChIP (chromatin immunoprecipitation) analyses revealed that YAP was recruited by *Wnt5a-1* gene promoter and increased Wnt5a expression. These results suggested that YAP responds to mechanical stress in hypertrophic adipocytes to induce the expression *Wnt5a*. When 8-week-old Wnt5a-FKO mice were fed an HFD for 20 weeks, the fat mass increased, especially in subcutaneous WATs, as compared with that observed in floxed mice, without significant changes in food intake or activity. Furthermore, Wnt5a-FKO mice showed impaired glucose tolerance regardless of diet type. Our findings show that hypertrophy/YAP/Wnt5a signaling constitutes a negative-feedback loop that retrains adipose tissue hypertrophy.

## Introduction

Epidemiological studies have demonstrated that obesity predisposes type 2 diabetes, cardiovascular diseases, and cancers of the pancreas, breast, colon, and liver [1]. However, mechanistic connections between obesity and chronic diseases remain unclear. Obesity is caused by the accumulation of white adipose tissue (WAT), which functions as an energy reservoir and endocrine organ that secretes many adipokines such as leptin, resistin, adiponectin, and TNFα [2, 3]. Adipose tissue is composed of various types of cells, including adipocytes, preadipocytes, mesenchymal stem cells, endothelial cells, and a variety of immune cells. During development and in the background of obesity, the numbers and sizes of adipocytes increase, which cause hyperplasia and adipocyte hypertrophy, respectively [3]. Adipocyte hypertrophy is mainly observed in the adipose tissues of the obese and is associated with inflammation.

This study shows Wnt5a might be a novel adipokine released by hypertrophic adipocytes to blood and suggests Wnt5a provides a molecular connection between obesity and obesity-related diseases in remote organs. Wnt5a is present in serum and its serum levels are correlated with the presence of chronic diseases, such as atherosclerosis, obesity, and rheumatoid arthritis, which suggests Wnt5a has endocrine functions [4–6]. Wnt ligands activate several signaling pathways, which are divided into two categories, canonical Wnt ligands that activate the transcriptional activity of β-catenin, and non-canonical Wnt ligands that activate other pathways, such as the planar cell polarity (PCP) and Wnt/Ca^2+^ pathways [7]. Wnt5a pathways are associated with several human pathologies, such as cancer, fibrosis, and inflammation, and the developments of male and female reproductive systems [8, 9]. Wnt5a functions as a mitogen and is required for the proliferation of mesodermal progenitors during development. The Wnt5a/PCP and Wnt5a/Ca^2+^ pathways are also associated with cell migration, which requires reorganization of the cytoskeleton and adhesion between cells and matrices [10–12]. Wnt5a was first identified as a target candidate for therapeutic intervention in inflammatory diseases based on findings that its serum levels were significantly increased in patients with severe sepsis or rheumatoid arthritis [13, 14] and that Wnt5a/FZD-5/CaMKII and Wnt5a/PCP/JNK signaling pathways were essential for activating inflammatory signals in macrophages [6, 13]. Furthermore, obese mice have lower serum levels of secreted frizzled-related protein 5 (Sfrp5), which sequesters Wnt5a in the extracellular space to prevent Wnt5a binding to its various receptors [15]. These observations indicate that the balance between Wnt5a and Sfrp5 controls degrees of obesity-related inflammation and insulin sensitivity [15].

In this study, we sought to identify the cellular source of Wnt5a secretion into the sera of obese mice and the mechanism underlying obesity-associated increases in Wnt5a expression. In adipocytes, Wnt5a knockout showed that adipocytes are the major cellular source of Wnt5a, and in C57BL/6 J mice fed a high-fat diet (HFD), hypertrophic adipocytes in obese fat tissues were filled with enlarged lipid droplets, which pushed cellular organelles and the cytoskeleton to cortical regions. YAP, a transcriptional co-activator and its paralog TAZ have been identified as mechano-transducers, which activate their target genes in response to changes in cytoskeletal tension. As transcriptional coactivators, active YAP and TAZ translocate to the nucleus to be recruited by the transcription factor TEAD to induce genes involved in cell proliferation, DNA replication, and the reinforcement of mechanical tension [16]. The current study shows that hypertrophic adipocytes increase Wnt5a expression by activating YAP. Furthermore, specific ablation of Wnt5a in adipocytes increased fat mass and glucose intolerance but did not downregulate the expressions of inflammatory cytokines in adipose tissues, which suggests that hypertrophy/YAP/Wnt5a signaling in adipocytes constitutes a negative feedback circuit that restrains the overexpansion of adipose tissues.

## Materials and methods

### Materials and antibodies

Insulin, dexamethasone (Dex), 3-isobutyl-1-methylxanthine (IBMX), Hoechst 33258, and Oil Red-O were purchased from Sigma-Aldrich (St. Louis, MO, USA). Anti-lamin A/C antibody was from Cell Signaling Technology (Danvers, MA, USA), anti-FLAG (900013) antibody from Gene Script (Piscataway, NJ, USA), anti-YAP antibody (SC101199) was from Santa Cruz Biotechnology (Dallas, TX, USA), and anti-β-actin (AC-15) was from Sigma-Aldrich (St. Louis, MO, USA). Anti-Wnt5a (MAB645) and anti-14-3-3γ (MAB5700) antibodies were obtained from R&D Systems (Minneapolis, MN, USA) and BODIPY493/503 (D3922) and fluorescent phalloidin conjugate (A34055) from Invitrogen (Waltham, MA, USA). cDNAs of Flag-YAP-5SA were subcloned into the pBABE retroviral vector. Normal diet (ND) (3.28 kcal/ g containing 14% of calories from fat) was purchased from Altromin Spezialfutter GmbH & Co. KG. (Lage, Germany) and the high-fat diet (HFD) (5.24 kcal/g containing 60% of calories from fat) from Research Diets, Inc. (New Brunswick, NJ, USA). Recombinant human IL‐6, IL-1β, and TNF-α were purchased from Peprotech (Saint Paul, MN, USA), and ELISA kits (OKEH04420) from AVIVA (San Diego, CA, USA).

### Experimental animals

All mice had a C57BL/6 J background. Eight-week-old male C57BL/6 J mice were fed either the ND or HFD for 8 or 35 weeks. Mice were maintained in a controlled environment (22 °C) under a 12 h light-dark cycle. All experiments were approved by and complied with the guidelines and regulations of Institutional Animal Care and Use Committee (UOS IACUC) (Permit Number: UOS-170516-1). Subcutaneous white adipose tissues (WATs) were isolated from inguinal fat depots attached dorsally along the pelvis and from epididymal WATs adjacent to gonads [17]. Fat specific *Wnt5a* knock-out mice (FKO) were generated by disrupting exon 2 of the *Wnt5a* gene. FKO mice were produced by crossing *Wnt5a*^*fl/fl*^ mice (Floxed, B6;129S-*Wnt5a*^*tm1.1Krvl*^/J) with Adiponectin-Cre mice (B6;FVB-Tg1(adipoq-Cre)Evdr/J) were obtained from the Jackson Laboratory (Bar Harbor, ME, USA). To test FKO, genomic DNA (gDNA) was extracted from the liver, subcutaneous WAT, small intestine, colon, and hind limb muscle of floxed and FKO mice using the Accu Prep^®^ Genomic DNA Extraction Kit (Bioneer, Daejeon, Korea). gDNA (100 ng) was used for PCR analysis. PCR primers were designed to amplify the genomic region covering two loxP sites flanking exon 2 of the *Wnt5a* gene on chromosome 14 [18]. Floxed and FKO mice (8 weeks old) were fed with ND or HFD for 20 weeks and maintained under a 12-h light/dark cycle at 22–24 °C in a specific pathogen-free facility with free access to water. All experiments were performed according to the “Guide for Animal Experiments” issued by the Korean Academy of Medical Sciences and were approved beforehand by the Institutional Animal Care and Use Committee of Seoul National University (Permit Number: SNU-151023-1-7).

### Indirect calorimetry, biochemical assays, and body compositions

Food intakes, activities, and oxygen consumptions (VO_2_) were measured using an indirect calorimetry system (TSE Systems, Bad Homburg, Germany). Chamber environments were maintained at a constant 22–24 °C. Serum triglyceride, total cholesterol, high-density lipoprotein (HDL), and cholesterol concentrations were determined using a Fuji Dry-Chem NX500i analyzer (FUJIFILM, Japan). Body compositions was analyzed by nuclear magnetic resonance (Minispec LF-50, Bruker, Germany).

### Intraperitoneal glucose tolerance test (IPGTT)

Glucose tolerance was assessed in mice using an IPGTT, which was performed by injecting 1.5 g/kg D-glucose (G8270, Sigma-Aldrich, MO, USA) intraperitoneally into overnight-fasted mice. Tail blood was collected before and 0, 15, 30, 60, 90, and 120 min after glucose administration. Blood glucose concentrations were measured using an Accu-Check glucose analyzer (Roche Diagnostics Ltd., Mannheim, Germany). Body weights, fasting glucose levels, and area under the IPGTT curve were analyzed using two-sample *t*-test. Data are presented as means ± standard errors of means, and statistical significance was accepted for P values <0.05.

### Cells

3T3-L1 mouse preadipocytes (ATCC, Rockville, MD, USA) were maintained and differentiated into mature adipocytes as previously described [19]. Cells were exposed to hypoxia (37 °C, 0.5% O_2_, 5% CO_2_, 95% N_2_) using a CO_2_ incubator (Water Jacketed CO_2_ incubator, Thermo Fisher Scientific, Waltham, MA, USA) and an anaerobic incubator (Model 1029; Forma Scientific, Inc., Marietta, OH, USA). YAP-5SA cells and EV cells were generated by infecting mouse 3T3-L1 cells with retroviruses encoding FLAG-tagged YAP-5SA or an empty vector, respectively, using the pBABE retroviral vector system and HEK293-based packaging cells (AmphoPack^TM^ 293 cell line) [20].

### Isolation of adipocytes and stromal vascular fractions

Adipocyte fractions and stromal vascular fractions (SVFs) were obtained by digesting subcutaneous WATs using collagenase, as previously described with some modification [21]. Chopped adipose tissue (1–2 g) was digested with 2 mg/ml collagenase I (Sigma, St. Louis, MO, USA) for 1 h at 37 °C with agitation at 150 rpm in 4 ml of KRBH buffer (120 mM NaCl, 4 mM KH_2_PO_4_, 1 mM MgSO_4_, 10 mM NaHCO_3_, 1 mM CaCl_2_, 30 mM HEPES (pH 7.4), 200 nM Adenosine and 1% bovine serum albumin). Mature adipocytes and SVFs were separated by centrifugation at 250× *g* for 5 min. The floating layer, which contained debris and mature adipocytes, was filtered through a 250 µm pierce tissue strainer (Thermo Fisher Scientific, Waltham, MA, USA) to remove tissue debris, and mature adipocytes were then removed from this layer using a pipette with a wide bore tip, and the pellet obtained containing SVF was resuspended in KRBH. The cell suspension was then filtered through a 100 μm cell strainer (Corning, NY, USA), and the filtrate was centrifuged at 250 ×g for 5 min. Cell pellets obtained in this manner are referred to as SVFs.

### Quantitative real-time reverse transcription-polymerase chain reaction (qRT-PCR) and Chromatin immunoprecipitation (ChIP) analysis

ChIP analyses were performed as previously described with some modifications [22]. After cross-linking, nuclei were isolated, and sonicated to shear chromatin using a Bioruptor Pico sonicator (Diagenode, a Hologic company, Leige, Belgium). DNA concentrations in the chromatin supernatants were measured to ensure that an equal amount of chromatin (20 μg of soluble chromatin) was used for immunoprecipitation with 2 μg of anti-YAP antibody at 4°C overnight. Primers for the *Wnt5a* and *Ctgf* genes used for ChIP analyses are detailed in Supplementary Table S2. qRT-PCR was performed using SYBR Green PCR master mix (Applied Biosystems, USA) and the QuantStudio™ 3 Real-Time PCR System (Applied Biosystems, USA). The primer sets for qRT-PCR and ChIP-PCR are listed in Tables S1 and S2.

### Knock-down using siRNA

3T3-L1 cells were transfected with siRNAs targeting YAP (CUG CUA UGA UAA CUA CGU U (Sense), AAC GUA GUU AUC AUA GCA G (AntiSense) and TAZ (CAC CAA CAG UAG CUC AGA U (Sense), AUC UGA GCU ACU GUU GGU G (AntiSense)) using Lipofectamine RNAi Max transfection reagent (Invitrogen, Thermo Fisher Scientific, Waltham, MA, USA). siRNAs and non-targeting siRNAs (AccuTarget^TM^ Fluorescein-labeled Negative Control) were purchased from Bioneer, Korea.

## Results

### Adipocyte-specific Wnt5a expression in obese mice

**Fig. 1:**
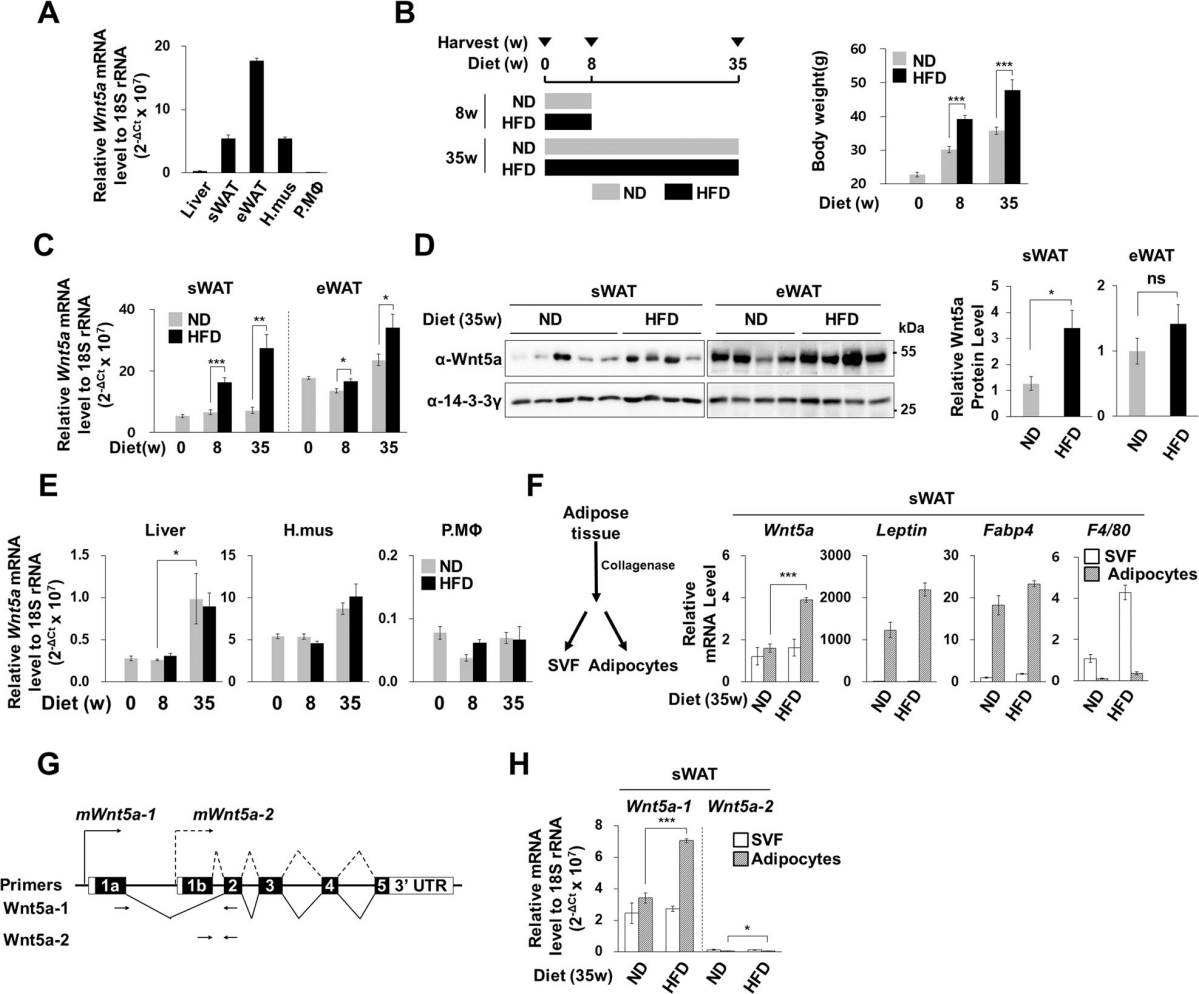
Wnt5a expression in obese mice. **A** Quantitative RT-PCR (qRT-PCR) of *Wnt5a* mRNA levels relative to 18 S rRNA levels in liver, subcutaneous white adipose tissue (sWAT), epididymal white adipose tissue (eWAT), hindlimb muscle (H.mus), and peritoneal macrophages (P.MΦ) of eight-week-old male C57BL/6 J mice (*n* = 4). The y-axis indicates *Wnt5a* mRNA levels relative to endogenous 18 S rRNA. The values indicate 2^−ΔCt^ times 10^7^ (ΔC_t_ = C_t Wnt5a_ − C_t 18S rRNA_; C_t_, qPCR threshold cycle defined by the ABI 7000 Real-Time PCR System), as previously described [26]. **B** Diet schedule and body weight. Eight-week-old male C57BL/6 J mice were fed a normal chow diet (ND) or a high fat diet (HFD) for the indicated periods: 0 w, *n* = 4; 8 w, ND, *n* = 6; 8 w, HFD, *n* = 6; 35 w, ND, *n* = 4; and 35 w, HFD, *n* = 4. **C** qRT-PCR of *Wnt5a* mRNA levels relative to 18 S rRNA levels in sWATs and eWATs. **D** Representative Western blot analyses of Wnt5a protein in sWAT and eWATs. 14-3-3γ was used as the loading control. Relative intensities of Wnt5a protein in sWAT (except an outlier sample in 3rd lane) and eWAT as determined by western blot (right graphs). **E** qRT-PCR of *Wnt5a* mRNA levels relative to 18 S rRNA levels in livers, hindlimb muscles (H.mus), and peritoneal macrophages (P.MΦ). **F** Adipocytes and SVFs were separated from sWATs as described in Materials and Methods (left diagram). qRT-PCR of the indicated genes in stromal vascular fractions (SVFs) or adipocytes isolated from sWATs. **G** Exons (black boxes) and untranslated regions (open boxes) of the *Wnt5a* gene on mouse chromosome 14. Solid and dotted lines indicate splicing regions and promoters of the *Wnt5a-1* and *Wnt5a-2* isoforms, respectively. Arrows indicate the primer sets used to determine the mRNA levels of each *Wnt5a* isoform (Supplementary Table S1). **H** qRT-PCR of *Wnt5a-1* and *Wnt5a-2* mRNAs in stromal vascular fractions (SVFs) or adipocytes isolated from sWATs. Statistical differences were assessed using a two-tailed paired Student’s *t*-test. *p < 0.05, **p < 0.01, ***p < 0.001; ns, not significant.

*Wnt5a* mRNA levels were quantified in several tissues of eight-week-old male C57BL/6 J mice. Subcutaneous and epididymal WATs and hindlimb muscles expressed relatively high *Wnt5a* mRNA levels as compared with livers and peritoneal macrophages, and epididymal WATs expressed three times more *Wnt5a* mRNA than subcutaneous WATs (Fig. 1A). C57BL/6 J mice were fed a normal diet (ND) or a high-fat diet (HFD) for 8 or 35 weeks (Fig. 1B). HFD increased Wnt5a mRNA and protein levels in subcutaneous WATs more so than in epididymal WATs (Fig. 1C, D), and HFD for 35 weeks increased *Wnt5a* expression more than HFD for 8 weeks (Fig. 1C). However, HFD did not increase *Wnt5a* expression in livers, hindlimb muscles, or peritoneal macrophages, although an age-dependent increase in *Wnt5a* mRNA was observed in liver tissues (Fig. 1E). To identify the cells responsible for the HFD-induced Wnt5a expression in subcutaneous WATs, we isolated adipocytes from stromal-vascular fractions (SVFs). Adipocyte-specific genes, such as *Fabp4* and *Leptin*, were only expressed in adipocyte fractions, while macrophage-specific *F4/80* was expressed in SVFs but not in adipocytes (Fig. 1F). *Wnt5a* was expressed in adipocytes and SVFs to similar extents in ND mice, and HFD increased its mRNA levels in adipocytes, but not in SVFs. We found that among two *Wnt5a* isoforms, *Wnt5a-1* and *Wnt5a-2*, which use different promoters and first exons, *Wnt5a-1* was the major isoform which HFD induced in adipocytes (Fig. 1G, H).

### Hypoxia and inflammatory cytokines did not increase Wnt5a expression

**Fig. 2:**
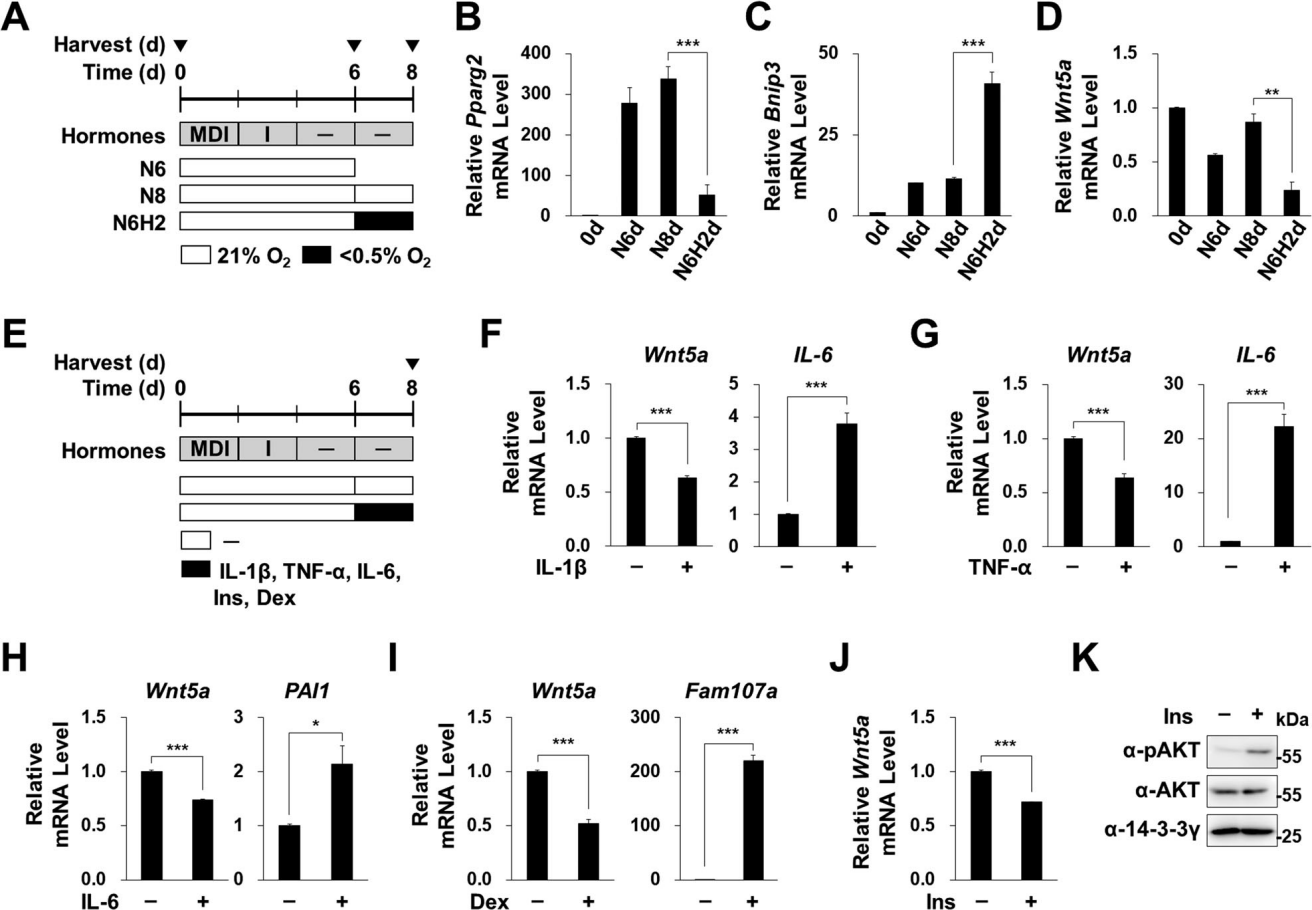
Effects of hypoxia and inflammatory cytokines on Wnt5a expression. **A** Postconfluent 3T3-L1 preadipocytes were induced to undergo adipogenesis by treating them with adipogenic hormones (IBMX (M), dexamethasone (**D**), and insulin (**I**)) as previously described [19] for 6 days, the mature adipocytes were then cultured under normoxia or hypoxia (H, < 0.5% O_2_) for 2 days. Cells were harvested at the indicated times after induction. **B**–**D** qRT-PCR analyses of *Pparg2*, *Bnip3*, and *Wnt5a* mRNA levels normalized versus 18 S rRNA levels. **E** Mature adipocytes were treated with IL-1β (40 ng/ml), IL-6 (30 ng/ml), TNF-α (40 ng/ml), insulin (Ins, 1 μM), or dexamethasone (Dex, 2 μM) for 2 days. **F**–**J** qRT-PCR analyses of *Wnt5a*, *IL-6, PAI1*, and *Fam107a* mRNA levels, which were normalized versus 18 S rRNA levels. IL-6, PAI1, and Fam107a mRNA levels were used as positive controls for each treatment. **K** Western blot analyses of phosphorylated AKT, total AKT, and 14-3-3γ proteins in insulin-treated 3T3-L1 preadipocytes, as described in Fig. 2E. 14-3-3γ was used as the loading control. qRT-PCR data are presented as means ± SEs. At least two independent experiments were performed. Statistical differences were assessed using a two-tailed paired Student’s t-test. *p < 0.05, **p < 0.01, ***p < 0.001; ns, not significant.

To study the mechanism whereby obesity increases Wnt5a expression in adipocytes, we generated mature adipocytes by differentiating 3T3-L1 preadipocytes with adipogenic hormone [19]. The O_2_ concentration in adipose tissues of obese mouse had been estimated 1.5~2 %, while 5~8 % in adipose tissue of lean mouse [23]. Hypoxic condition in obese adipose tissues induces many inflammatory cytokines and reduced adipogenesis in adipose tissues [20]. Hypoxia and chronic inflammation are characteristics of obese WATs and cause insulin resistance in adipocytes [24, 25]. We exposed mature adipocytes to hypoxia for 2 days (Fig. 2A). We confirmed that adipogenic hormone treatment induce a master adipogenic transcription factor, *Pparg2*, and that this induction was repressed by hypoxic exposure (Fig. 2B) [20, 26]. Hypoxia increased the expression of the hypoxic target gene, *Bnip3*, but decreased *Wnt5a* expression (Fig. 2C, D). Next, we treated mature adipocytes with several inflammatory cytokines, such as IL-1β, IL-6, and TNF-α (Fig. 2E) [27], and although these cytokines increased the expressions of their target genes, *IL-6* and *PAI-1* [28, 29], they decreased *Wnt5a* expression (Fig. 2F–H). Glucocorticoid and insulin levels have been reported to be elevated in obese individuals [30, 31], and we observed dexamethasone (a glucocorticoid agonist) induced a well-known target gene, *Fam107a*, but reduced *Wnt5a* levels (Fig. 2I). Insulin also reduced *Wnt5a* mRNA levels in adipocytes, but increased phosphorylation of Akt (S473) which is an indicator of insulin treatment (Fig. 2J, K). These results indicate that obesity-induced signals, that is, hypoxia, IL-1β, IL-6, TNF-α, glucocorticoid, or insulin, did not increase *Wnt5a* expression in adipocytes.

### Adipocyte hypertrophy induced Wnt5a expression through YAP activation

**Fig. 3:**
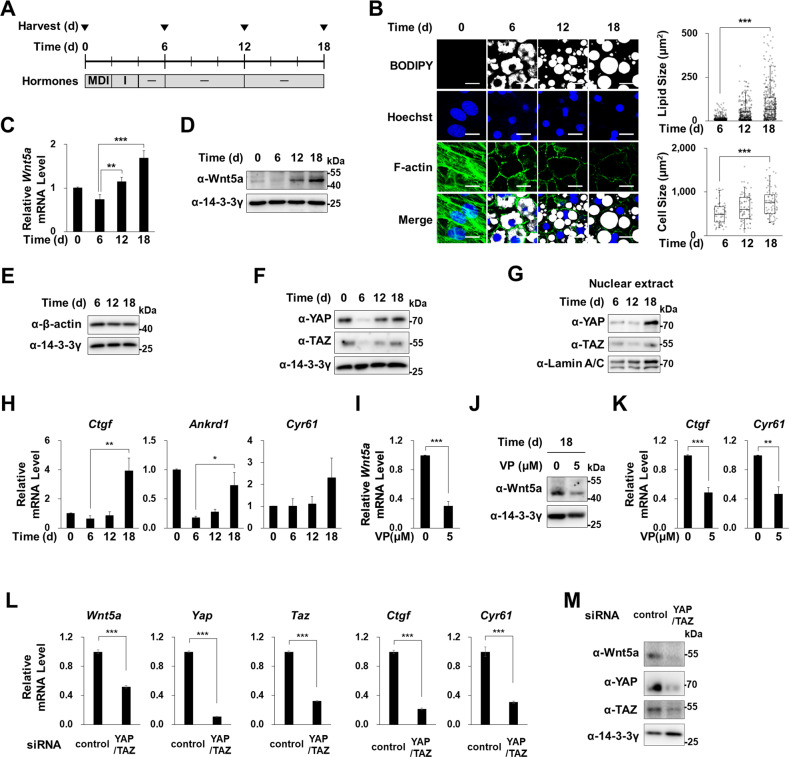
Effects of adipocyte hypertrophy on Wnt5a expression. **A** Mature adipocytes were further cultured for 6 or 12 days and then harvested at the indicated times after induction. **B** Confocal microscopic images of F-actin (green), lipid vacuoles (white), and nuclei (blue) visualized by staining with Phalloidin-Alexa555, BODIPY 493/503, or Hoechst 33258, respectively (left panel). Sizes of lipid vacuoles and cells were measured in BODIPY stained areas and F-actin covered areas, respectively, using ImageJ. 58~74 cells were observed at each time point. Scale bars indicate 20 μm. **C** qRT-PCR analyses results of *Wnt5a* mRNA levels expressed as means ± SEs. All experiments were repeated independently at least twice. **p < 0.01, and ***p < 0.001 by a Student’s *t*-test. **D**–**F** Western blot of Wnt5a, β-actin, YAP, and TAZ for whole lysates of adipocytes cultured and harvested as described in Fig. 3A. 14-3-3γ was used as the loading control. **G** Western blot analyses of YAP and TAZ in nuclear fraction of mature adipocytes obtained as shown Fig. 3A. Lamin A/C was used as the loading control for the nuclear fraction. **H** qRT-PCR of *Ctgf, Ankrd1* and *Cyr61* mRNA levels. **I**–**K** Mature adipocytes were treated at 18 days after adipogenic induction with Verteporfin (VP; 5 μM) for 4 h and harvested. **I** qRT-PCR of *Wnt5a* mRNA (**J**) Western blot analyses of Wnt5a protein, (**K**) qRT-PCR of *Ctgf* and *Cyr61* mRNA levels (normalized versus 18 S rRNA levels). **L** qRT-PCR of *Wnt5a, Yap, Taz, Ctgf*, and *Cyr61* (**M**) Western blot analyses of Wnt5a, YAP, TAZ proteins in 3T3-L1 preadipocytes transfected with control siRNA or siRNAs against YAP and TAZ. qRT-PCR results are presented as means ± SEs. Experiments were conducted independently at least twice. The significances of differences were determined using a two-tailed paired Student’s t-test. *p < 0.05, **p < 0.01, ***p < 0.001; ns, not significant.

Hypertrophy in adipocytes is associated with inflammation, metabolic dysregulation, and the secretions of adipokines [32]. To mimic adipocyte hypertrophy in vitro, we cultured mature adipocytes for an additional 6 or 12 days after 6 day adipocyte differentiation (Fig. 3A), which further increased lipid droplet and adipocyte sizes (Fig. 3B). We found that these hypertrophic changes in adipocytes co-occurred with increases in Wnt5a mRNA and protein levels (Fig. 3C, D). It has been previously reported that large lipid droplets in adipocytes cause cytoskeletal and nuclear rearrangements in hypertrophic adipocytes [33]. We observed by time course staining with phalloidin (a fluorescent probe specific for filamentous actin (F-actin)) cytoplasmic F-actin stress fiber were reduced and that fibers were reorganized only at the cortical region during adipogenesis (Fig. 3B). Culture for an additional 6 or 12 days reduced F-actin even at the cortical region but without reducing total β-actin protein levels (Fig. 3E). These mechanical changes that occur during the development of hypertrophy can regulate the mechanotransducer YAP and TAZ, which by acting as a transcriptional coactivator translates physical changes into cell-specific transcription [34, 35]. We observed adipogenesis reduced YAP and TAZ protein levels, but that culture for an additional 6 or 12 days revived these levels, especially in the nuclei of hypertrophic adipocytes (Fig. 3F, G). Furthermore, the mRNA levels of genes targeted by YAP/TAZ, that is, connective tissue growth factor *(Ctgf)*, Ankyrin repeat domain 1*(Anrkd1)*, and Cysteine-rich angiogenic inducer 61 (*Cyr61)* consistently increased in hypertrophic adipocytes (Fig. 3H). Verteporfin (VP) is a pharmacological inhibitor that represses YAP/TAZ coactivator-activity by preventing it from associating with its partner transcription factors (TEA domain transcription factors, TEADs) [36]. Treatment of hypertrophic adipocytes with VP reduced Wnt5a mRNA and protein levels (Fig. 3I, J), and reduced the expressions of YAP target genes (*Ctgf* and *Ankrd1*) (Fig. 3K). Furthermore, siRNA knock-down of YAP and TAZ reduced the mRNA levels of their target genes (*Ctgf* and *Cyr61*) and reduced Wnt5a mRNA and protein levels (Fig. 3L, M). These results suggest that hypertrophy induces Wnt5a expression by activating the mechanosensor YAP/TAZ in adipocytes.

### Active YAP induced Wnt5a expression

**Fig. 4:**
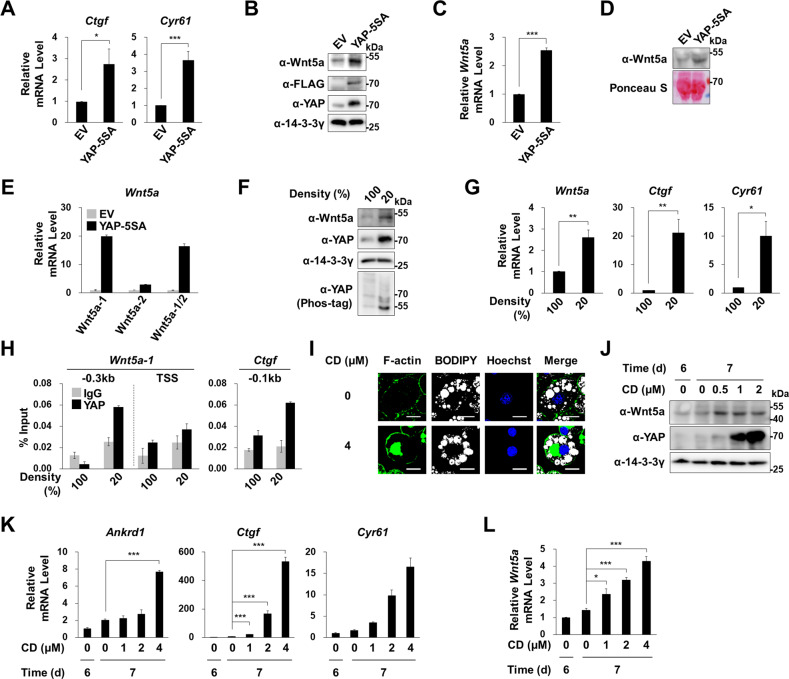
YAP activation and Wnt5a expression. **A**–**E** 3T3-L1 cells were infected with a retrovirus encoding FLAG-tagged YAP-5SA (YAP-5SA) or empty vector (EV). **A** qRT-PCR analyses of *Ctgf* and *Cyr61* mRNA levels, which were normalized versus 18 S rRNA levels. **B** Western blot analyses using antibodies against Wnt5a, FLAG, YAP, and 14-3-3γ. **C** qRT-PCR analyses of *Wnt5a* mRNA levels, which were normalized versus 18 S rRNA levels (**D**) Western blot analyses of Wnt5a in conditioned media (CM) harvested from EV- or YAP-5SA-infected 3T3-L1 cells cultivated for 2 days after seeding. Ponceau S staining was performed to confirm equal loadings. **E** qRT-PCR using specific primer sets for *Wnt5a-1* or *Wnt5a-2* mRNA isoforms or both isoforms. **F** Western blot analyses of Wnt5a, YAP, and 14-3-3γ proteins in 100% and 20% confluent 3T3-L1 cells. 14-3-3γ was used as the loading control. Phosphorylated YAP was separated by Phos-tag^TM^-containing SDS-PAGE. **G** qRT-PCR of *Wnt5a, Ctgf*, and *Cyr61* mRNA levels in 100% and 20% confluent 3T3-L1 cells. **H** ChIP-qPCR analyses of YAP occupancy of the indicated regions from the TSSs of the *Wnt5a* or *Ctgf* genes in 100% or 20% confluent 3T3-L1 cells [19]. **I**–**L** Mature adipocytes obtained 6 days after adipogenic induction were treated with Cytochalasin D (CD; 4 μM) for 1 day. **I** Confocal microscopic images of cellular F-actin (green), lipid vacuoles (white), and nuclei (blue) visualized by staining with Phalloidin-Alexa555, BODIPY 493/503, or Hoechst 33258, respectively. Scale bars indicate 20 μm. (**J**) Western blot analyses of Wnt5a and YAP. **K**, **L** qRT-PCR of *Ankrd1, Ctgf, Cyr61*, and *Wnt5a* mRNAs. qRT-PCR data are presented as means ± SEs. At least two independent experiments were conducted. The significances of differences were assessed using a two-tailed paired Student’s t-test. *p < 0.05, **p < 0.01, ***p < 0.001; ns, not significant.

To investigate the nature of the link between YAP activation and Wnt5a induction, we assessed changes in Wnt5a expression in cells containing activated YAP. We overexpressed an active mutant YAP (YAP-5SA), in which five serine residues (S61, S109, S127, S164, S381) were replaced with alanine residues [37]. Overexpression of YAP-5SA increased *Ctgf* and *Cyr61* mRNA levels (Fig. 4A) and Wnt5a mRNA and protein (Fig. 4B, C) levels. Western blot of conditioned media (CM) revealed that YAP-5SA overexpressing cells secreted more Wnt5a protein (Fig. 4D). As was observed in obese adipose tissues (Figs. 1G, H), YAP-5SA increased *Wnt5a*-1 gene expression (Fig. 4E), which suggested that YAP regulates *Wnt5a-1* gene promoter but not *Wnt5a-2* gene promoter.

In sparse cells, actomyosin activity promotes the nuclear location of YAP, whereas in confluent cells it attenuates YAP protein levels [37]. Western blot showed that both Wnt5a expression and YAP activity were greater in sparse 3T3-L1 cells than in confluent cells (Fig. 4F, G). ChIP-qPCR analyses of sparse cells showed YAP was recruited at the –0.3 kb region from the transcription start site of the *Wnt5a-1* gene, and that YAP was located on the promoter region of the *Ctgf* gene (a positive control gene) (Fig. 4H). Cytochalasin D (CD) is mycotoxin known to block F-actin assembly by binding to the barbed end where actin polymer is elongated [38]. Interestingly, CD treatment dramatically rearranged F-actin from a cortical to a disorganized tangled structure in mature adipocytes (Fig. 4I), and this dramatic rearrangement of F-actin promoted the nuclear localization of YAP [39]. In mature adipocytes, CD treatment increased the levels of YAP and its target genes*, Ankrd1, Ctgf*, and *Cyr61* (Fig. 4J, K) and dose-dependently increased Wnt5a mRNA and protein levels (Fig. 4J–L). These results show that active YAP increases *Wnt5a-1* gene expression.

### YAP activity in obese subcutaneous WATs

**Fig. 5:**
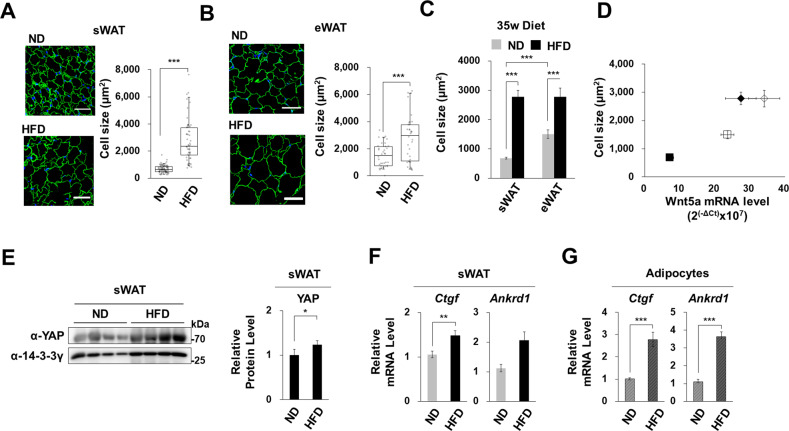
YAP activation in the adipocytes of HFD fed mice. **A**–**D** Subcutaneous WAT (sWATs) and epididymal WATs (eWATs) of male C57BL/6 J mice fed ND or HFD for 35 weeks from 8 weeks old. **A**, **B** Confocal microscopic images of cellular FABP (green) and nuclei (blue) in subcutaneous and epididymal WATs, respectively (left panel). **C** Cell sizes (μm^2^) were determined from cellular FABP areas using ImageJ. 52~78 cells were observed for each diet. Scale bars indicate 50 μm. **D** Correlation between cell sizes and *Wnt5a* mRNA levels. The x-axis indicates *Wnt5a* mRNA levels relative to endogenous 18 S rRNA and the y-axis indicates adipocyte sizes (μm^2^) as observed in sWATs after 35 w of the HFD (◆), eWATs after 35 w of the HFD (◇), sWAT after 35 w of the ND (■), and eWAT after 35 w of the ND (□) mice (diet programs are shown in Fig. 1B). **E** Western blot analyses of Wnt5a, YAP, and 14-3-3γ proteins in sWATs of four male C57BL/6 J mice fed the ND or the HFD for 35 weeks from 8 weeks old. Relative intensities of YAP proteins in sWATs were assessed by western blot (right graphs). **F**, **G** qRT-PCR of *Ctgf* and *Ankrd1* mRNA levels in sWATs and adipocytes isolated from the sWATs of mice fed the ND or the HFD for 35 weeks from 8 weeks old. mRNA levels were normalized versus 18 S rRNA levels. qRT-PCR data are presented as means ± SEs. All experiments were conducted independently at least twice. The significances of differences were determined using a two-tailed paired Student’s t-test. *p < 0.05, **p < 0.01, ***p < 0.001; ns, not significant.

HFD increased the average size of adipocytes in subcutaneous and epididymal WATs by 4- and 2-fold, respectively (Fig. 5A to C). When fed the ND, adipocytes sizes in epididymal WATs were almost twice the size as those in subcutaneous WATs, but the HFD greatly increased adipocyte in subcutaneous WATs such that adipocyte sizes in subcutaneous and epididymal WATs were similar (Fig. 5C). Interestingly, adipocyte sizes and *Wnt5a* mRNA expression levels were positively correlated (Fig. 5D). HFD fed obese mice had higher levels of YAP and Wnt5a proteins in subcutaneous WATs than ND fed mice (Figs. 1D and 5E). Furthermore, the mRNA levels of *Ctgf* and *Ankrd1* (YAP target genes) were consistently correlated with adipocyte sizes in subcutaneous WATs (Fig. 5F), and the HFD increased the expression levels of YAP target genes (*Ctgf* and *Ankrd1*) in isolated adipocytes to a greater extent than in adipose tissues (Fig. 5F, G). These observations show that hypertrophy in adipocytes increased YAP activity, and thus, Wnt5a expression (its target gene) in obese adipose tissues.

### Adipocytes were the major source of Wnt5a in obese mouse sera

**Fig. 6:**
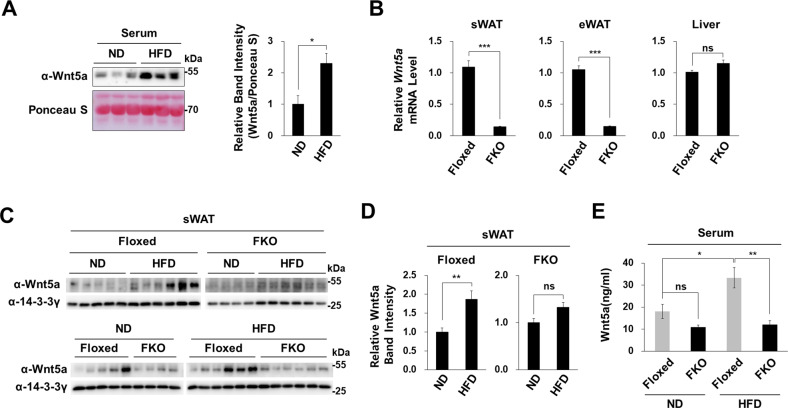
Obesity and Wnt5a expression in fat specific Wnt5a knock-out mice. **A** Western blot analyses of Wnt5a protein in mouse serum (left panel). Ponceau S staining was performed to confirm equal loadings. Serum was obtained from the hearts of three male C57BL/6 J mice fed the ND or the HFD for 20 weeks from 8 weeks old. Relative band intensities of Wnt5a were obtained using ImageJ software and normalized versus Ponceau S band intensities (right panel). **B**–**D** Fat specific *Wnt5a* knock out (FKO) mice were generated by disrupting *Wnt5a* exon 2, as described in Supplementary Fig. S1A. **B** qRT-PCR of *Wnt5a* mRNA levels normalized versus 18 S rRNA levels in liver, subcutaneous WATs, or epididymal WATs of male *Wnt5a*^*fl/fl*^ mice (Floxed), and male FKO mice fed the ND for 20 weeks from 8 weeks old (Floxed 20 w ND, *n* = 5; FKO 20 w ND, *n* = 4). **C** Western blot analyses of Wnt5a protein in the sWATs of Floxed and FKO mice fed the ND or the HFD for 20 weeks from 8 weeks old. 14-3-3γ was used as the loading control. **D** Relative band intensities of Wnt5a were determined using ImageJ software and normalized versus 14-3-3γ band intensities. **E** Concentration of Wnt5a protein in the sera of Floxed and FKO mice as determined using a Mouse Wnt5a ELISA kit (AVIVA). Floxed and FKO mice were fed the ND or the HFD for 20 weeks from 8 weeks old (Floxed ND, *n* = 5; Floxed HFD, *n* = 6; FKO ND, *n* = 4; FKO HFD, *n* = 6). The significances of differences were assessed using a two-tailed paired Student’s *t*-test. *p < 0.05, **p < 0.01, ***p < 0.001; ns, not significant.

In line with previous reported that obesity increases Wnt5a protein levels in the sera of humans and mice [40, 41], we found that serum Wnt5a protein levels were higher for HFD fed mice than ND fed mice (Fig. 6A). We examined whether adipocytes contributed to increased serum Wnt5a levels of obese mice by generating fat-specific *Wnt5a* knockout (FKO) mice (Supplementary Fig. 1A–C). Specific *Wnt5a* ablation significantly reduced *Wnt5a* mRNA levels in WATs but did not affect *Wnt5a* mRNA levels in liver tissues, which confirmed *Wnt5a* was specifically ablated in adipocytes (Fig. 6B and Supplementary Fig. 1C). Adipocyte-specific ablation of the *Wnt5a* gene did not significantly reduce Wnt5a protein levels in the subcutaneous WATs or sera of ND mice (Fig. 6C, D). These results showed that adipocytes in lean mice partially contributed to Wnt5a protein levels in adipose tissues and serum. Wnt5a was secreted by adipocytes and other cells in adipose tissues into the sera of lean mice (Fig. 6C, D and Supplementary Fig. S2). Furthermore, adipocyte-specific ablation of *Wnt5a* completely prevented the HFD increasing Wnt5a levels (Figs. 6C, D), indicating that adipocytes alone increased Wnt5a levels in obese mice. Thus, *Wnt5a* ablation significantly reduced Wnt5a protein in subcutaneous WATs and serum in HFD fed mice but not in ND fed mice (Fig. 6C, E).

### Fat-specific Wnt5a KO increased fat mass specifically in subcutaneous WATs

**Fig. 7:**
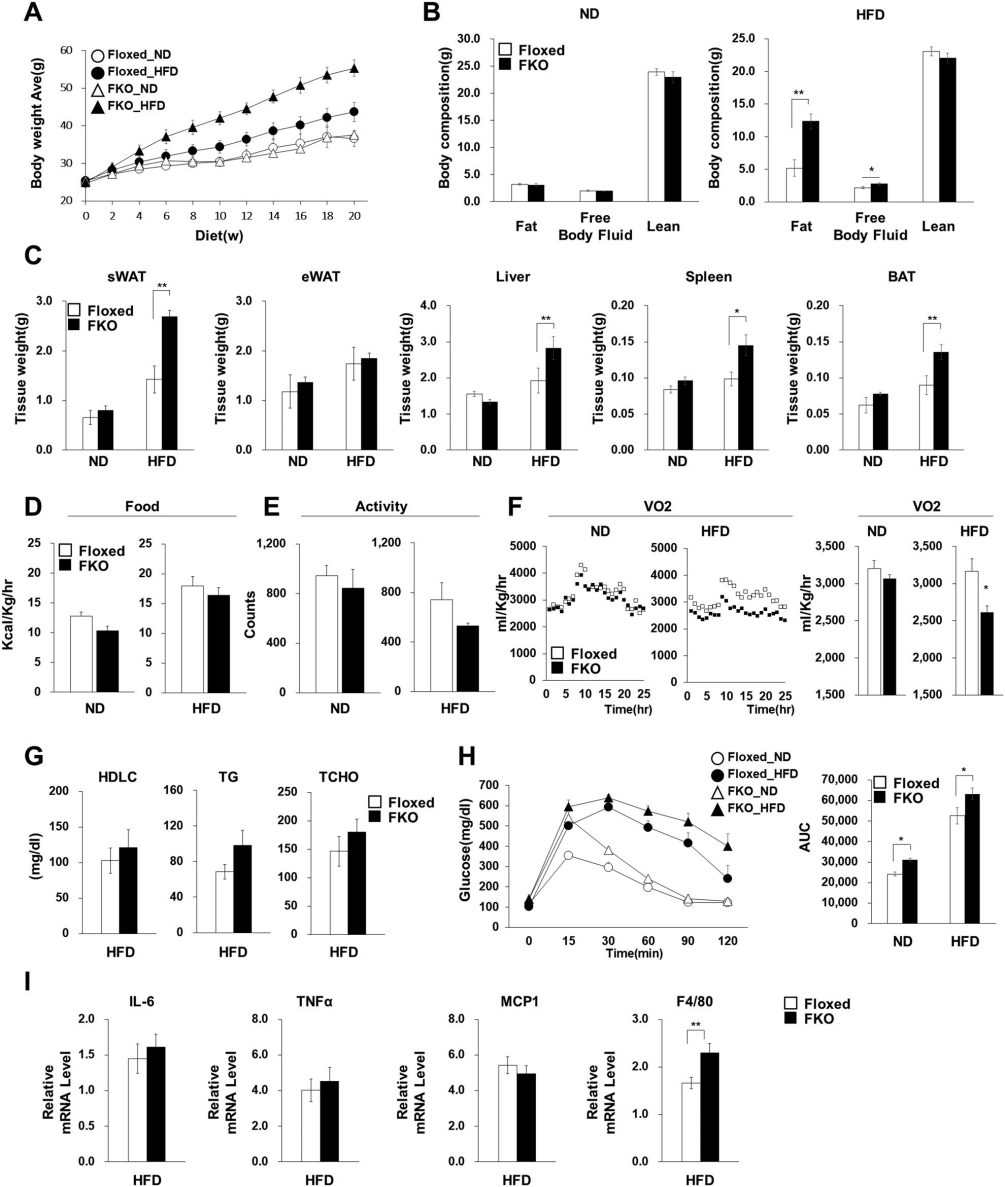
Wnt5a-FKO mice after 20 weeks on the HFD. Floxed and Wnt5a**-** FKO mice were fed the ND or the HFD for 20 weeks from 8 weeks old. **A** Comparison of the body weights of Floxed or FKO mice on the normal chow diet (ND) or the high fat diet (HFD) for the indicated periods. Mice were weighed at fortnightly (Floxed, ND, *n* = 4; FKO, ND, *n* = 3; Floxed, HFD, *n* = 6; FKO, HFD, *n* = 6). **B** The total fat, free body fluid, and lean body masses were analyzed by nuclear magnetic resonance (Floxed, ND, *n* = 4; FKO, ND, *n* = 4; Floxed, HFD*, n* = 6; FKO, HFD, *n* = 6) after 8 weeks on the ND or the HFD. (**C**) Weights of sWAT, eWAT, liver, spleen, and BAT of Floxed and FKO mice (Floxed, ND, *n* = 5; FKO, ND, *n* = 4; Floxed, HFD, *n* = 6; FKO, HFD, *n* = 6). **D**–**F** Food intakes (D), activities (**E**), and VO_2_ values (**F**) as determined by indirect calorimetry for Floxed and FKO mice (Floxed, ND, *n* = 4; FKO, ND, *n* = 4; Floxed, HFD, *n* = 6; FKO, HFD, *n* = 6) after 8 weeks on the ND or the HFD. (**G**) Serum biochemistry of lipid parameters in overnight-fasted Floxed and FKO mice (Floxed, HFD, *n* = 6; FKO, HFD, *n* = 6). (**H**) GTT and AUC analysis. Blood glucose concentrations of mice after 9 weeks on the ND or HFD were measured at various times after glucose (1.5 g/kg) treatment. Areas under the curve (AUC) for intraperitoneal glucose tolerance test results were calculated (Floxed, ND, *n* = 5; FKO, ND, *n* = 5; Floxed, HFD, *n* = 6; FKO, HFD, *n* = 7). **I** Relative mRNA expression levels of *IL-6, TNFα, MCP1*, and *F4/80* in sWATs of Floxed and FKO mice (Floxed, HFD, *n* = 6; FKO, HFD, *n* = 6). mRNA levels were normalized versus 18 S rRNA. Data are the means ± SEMs of three independent determinations. The significances of differences were determined using a two-tailed paired Student’s t-test. *p < 0.05, **p < 0.01.

To identify the effect of adipocyte-specific ablation of the Wnt5a gene, we first measured the body weights of ND and HFD fed mice during the 20-week feeding period. Wnt5a-FKO mice had normal body weights when fed the ND, but when fed the HFD, Wnt5a-FKO mice exhibited body weight increases of up to 126.8%, which was mainly due to higher fat contents (Fig. 7A, B). After 20 weeks on the HFD, Wnt5a-FKO mice had greater fat-containing organ weights such as for subcutaneous WAT, BAT liver, and spleen than floxed mice (Fig. 7C), whereas epididymal WAT weights were no greater than in floxed mice. Consistent with the findings that HFD increased adipocyte size and Wnt5a expression in subcutaneous WATs more than in epididymal WATs (Fig. 5A–D), these results indicate that Wnt5a ablation had a greater influence on weight gain in subcutaneous WATs. Compared to floxed mice, no significant change in food intake or activity was observed in Wnt5a-FKO mice fed the ND or the HFD (Fig. 7D, E), but metabolic rate, as determined by oxygen consumption (VO_2_), was lower in Wnt5a FKO-HFD fed mice (Fig. 7F).

To assess the systemic effect of increased adiposity in Wnt5a-FKO mice, we measured serum lipid parameters. Triglyceride and total cholesterol levels were not significantly greater in the HFD-fed Wnt5a-FKO mice than floxed mice (Fig. 7G). However, glucose tolerance testing showed Wnt5a-FKO mice showed impaired glucose tolerance under both ND and HFD conditions as compared with floxed mice (Fig. 7H). A report that Wnt5a signaling increases the expressions of several cytokines and promotes adipose tissue inflammation [6] led us to investigate whether Wnt5a-FKO changed the expressions of inflammatory cytokines (IL-6, TNF-α, MCP-1, and F4/80) in subcutaneous WATs, but no significantly effect was observed (Fig. 7I).

These results showed hypertrophic adipocytes are responsible for obesity-related increases in Wn5a levels in sera and that Wnt5a in obese adipose tissues functions as an autocrine or paracrine factor that inhibits the expansion of adipose tissues, which suggests hypertrophy/YAP/Wnt5a signaling constitutes a negative-feedback loop that prevents the overexpansion of adipose tissues.

## Discussion

This is the study first to show that adipocytes are mainly responsible for increased serum Wnt5a levels in obese mice and that adipocyte hypertrophy activates YAP/TAZ, which in turn, induces the expression of Wnt5a. In addition, we found subcutaneous WATs more sensitively responded to obesity in terms of increases in adipocyte size and Wnt5a induction than epididymal WATs. On the other hand, in human subjects obesity has been reported to be associated with *Wnt5a* mRNA production primarily in visceral adipose tissues [6, 41, 42]. Mouse models are excellent for studying human diseases, but human visceral fat tissues differ from those of mice. The human prototypical visceral depot is omental fat, an intra-abdominal depot lining the human stomach surface, and this is barely observed in mice. Conversely, epididymal WAT, located next to the gonads of male mice is the largest and most typical visceral depot in male mice but it is not equivalent to human omental fat due to several anatomical and physiological differences, which include blood circulatory differences [17]. Thus, mouse epididymal WAT is limited in terms of its ability to mimic human omental visceral fat. This study showed that obesity increases Wnt5a production in subcutaneous WAT more so than in epididymal WAT in mice, whereas in man production in omental visceral fat is greater than from in subcutaneous WAT [43–45].

We also found that the HFD for 35 weeks increased adipocyte sizes by 180 and 400% in epididymal and subcutaneous WATs, respectively (Fig. 5A, B), which suggests that adipocytes in subcutaneous WATs, but not in epididymal WATs, of obese mice continuously enlarge during HFD administration [44, 45]. Furthermore, adipocyte size was positively correlated with *Wnt5a* expression (Fig. 5D). In lean mice, the larger adipocytes in epididymal WATs expressed more *Wnt5a* than the smaller adipocytes in subcutaneous WATs, while in obese mice enlarged adipocytes in subcutaneous WATs were as large as adipocytes in epididymal WATs and expressed as much *Wnt5a* (Fig. 5D). In obese humans, Wnt5a expression in omental fat is greater than in subcutaneous fat, which contradicts our findings in mice [46–48]. However, human omental fat more resembles mouse subcutaneous WATs, for example, mouse subcutaneous WAT enlarges mainly because of hypertrophy induced by extended overfeeding as does human omental fat, and human subcutaneous fat and mouse epididymal fat similarly become enlarged due to hypertrophy and hyperplasia [43–45].

Interferometric phase microscopy revealed that lipid droplets in adipocytes are stiffer than in cytoplasm, which suggests lipid droplets mechanically distort internal environments [33, 49]. Furthermore, droplet stiffness increases with size and leads to cytoskeletal and nuclear rearrangements. Recently, YAP/TAZ was identified as a mechanotransducer that translates physical changes into cell-specific transcriptions. Flattening of a nucleus results in the asymmetric opening of nuclear pores and may preferentially promote the nuclear importation of YAP rather than its export [39]. In this study, we demonstrate that hypertrophy of adipocytes in obese mice activates YAP/TAZ, which then induces Wnt5a. Together with the findings that Wnt5a increases inflammation and fibrosis, our results imply a vicious cycle whereby hypertrophy-induced YAP/TAZ and Wnt5a increase the stiffness of ECM in obese adipose tissues, which further increases mechanical stress and chronic inflammation. Wnt5a and CTGF, a YAP target gene can cooperate with TGF-β to induce sustained fibrosis, [50] which is a hallmark of human obese WAT and causes obesity-induced chronic inflammation [51].

Our observation that Wnt5a-FKO mice fed the HFD, but not the ND, gained more fat mass than floxed mice fed the HFD (Fig. 7A–C) suggests that Wnt5a limited the over-expansion of adipose tissues, which is consistent with the finding of a previous study that identified genetic differences between an obesity-prone mouse strain (C57Bl/6 (B6)) and an obesity-resistant mouse strain (129S6/SvEvTac (129)) [52]. Genome-wide analyses of these two strains revealed that *Wnt5a* expression was significantly lower in B6 than in 129 mice. Furthermore, multi-dimensional single-cell analyses of fibro/adipogenic progenitors (FAPs) isolated from skeletal muscles consistently identified Wnt5a as a crucial autocrine/paracrine factor that restrained deleterious adipogenesis in injured muscles [53]. Furthermore, Wnt5a expression was significantly downregulated in FAP-isolated from *mdx* mice, an animal model of Duchenne muscular dystrophy, which is prone to adipogenesis in dystrophic muscles, and exogenous administration of Wnt5a prevented the in vitro adipogenesis of isolated FAPs from mdx mice, thus confirming that Wnt5a inhibits adipogenesis. Our finding that Wnt5a-FKO mice on the HFD gained more subcutaneous white adipose tissue fat mass than C57BL/6 J controls suggests that hypertrophy/YAP/Wnt5a signaling in obese fat cells constitutes a negative feedback control circuit that limits the over-expansion of fat tissues during HFD.

Wnt5a has been identified as an inflammatory factor that activates JNK to induce inflammatory cytokines, such as IL-6 and TNF-α. A previous study using myeloid-specific ablation of Wnt5a showed that by acting as an autocrine/paracrine factor, Wnt5a activates the JNK pathway to promote the expressions of several inflammatory cytokines in myeloid cells more effectively than in adipocytes [6, 15]. However, in contrast to its effects in myeloid cells, in FAP progenitor cells Wnt5a barely affected the phosphorylation of JNK instead increased the phosphorylation of the GSK3 inhibitory residue, and thus, stabilized β-catenin [53]. Thus in FAP progenitor cells, Wnt5a can activate canonical β-catenin-dependent signaling, which is known to repress PPARγ and inhibits adipogenesis. These cell-type-specific responses to Wnt5a probably depend on cell-specific receptor contexts [19, 53]. In the present study, we performed in vivo experiments on male mice without sex-matched controls. Several human studies that included age- and sex-matched controls did not detect a significant difference between males and females in terms of obesity-induced Wnt5a expression [40, 54]. Although Wnt5a is detected at high concentrations in the sera of obese humans and mice, it has not been determined whether by acting as an endocrine factor Wnt5a affects remote organs. This study is the first to report that adipocytes are the main source of cells that secrete additional amounts of Wnt5a in obese mice and that hypertrophy/YAP/Wnt5a signaling constitutes a negative-feedback loop that restrains the overexpansion of adipose tissues.

## Supplementary information

Supplemental Material

Checklist

## Data Availability

The data sets generated during this study are available from the corresponding author, Hyunsung Park, upon receipt of reasonable request.
