# Supplementary material for: YAP-dependent Wnt5a induction in hypertrophic adipocytes restrains adiposity
Source: Cell Death Dis. 2022 Apr 27;13(4):407. doi: 10.1038/s41419-022-04847-0 (PMC9046197; doi:10.1038/s41419-022-04847-0)
Supplement: Supplementary file 1 — Supplemental Material [file 41419_2022_4847_MOESM1_ESM.docx]

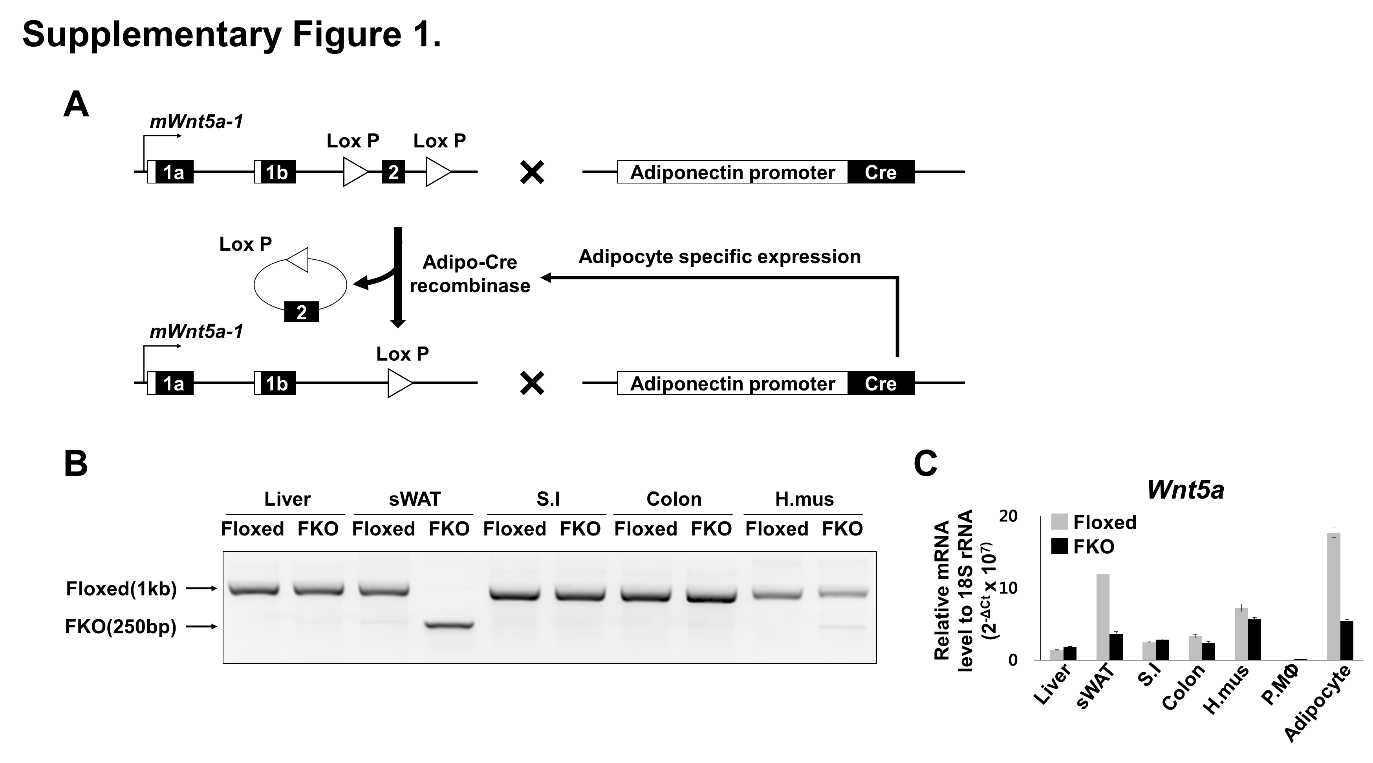


**Supplementary Figure 1. Fat-specific Wnt5a KO mice** (A) Schematic representation of the Cre-LoxP strategy used to generate Wnt5a-FKO. B6;129S-*Wnt5a^tm1.1Krvl^*/J has Lox P sites on both sides of Wnt5a Exon 2 (Floxed) and B6;FVB-Tg(Adiponectin-cre)1Evdr/J has the Cre gene which is regulated by the adiponectin promoter. (B) PCR of genomic DNA encoding *Wnt5a* Exon 2 in liver, sWAT, Small intestine (SI), Colon, and hind muscle (H. mus) of 8-week-old Floxed and FKO mice. Wnt5a floxed DNA fragment (1 kb) and Wnt5a knockout DNA fragment (250 bp) were detected. (C) qRT-PCR of *Wnt5a* mRNA normalized with 18S rRNA in liver, sWAT, small intestine (S.I), colon, hind muscle (H.mus), peritoneal macrophage (P.MΦ) and adipocytes from WAT of 13-week-old male Floxed and FKO mice (Floxed 20 w, ND, n=1; FKO 20 w, ND, n=1). The y-axis indicates the *Wnt5a* mRNA level relative to the endogenous 18S rRNA.

**
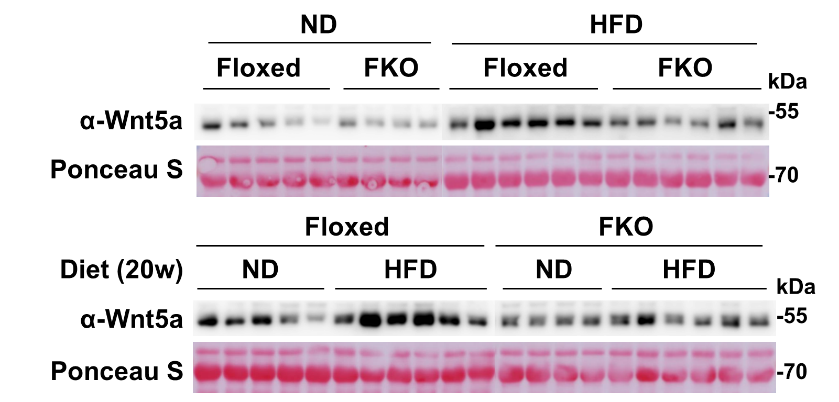
**

**Supplementary Figure 2. Wnt5a protein levels in fat-specific Wnt5a KO mice** Western blot analyses of the Wnt5a protein in sera of Floxed and FKO mice which were fed with either ND or HFD for 20 weeks since 8 weeks old.

**Supplementary Table S1. Primer sequences used in qRT-PCR analyses**.

| **Mouse Gene** | **Strand** | **Sequence** |
| --- | --- | --- |
| *18S rRNA* | Sense | ACCGCAGCTAGGAATAATGGAATA |
|  | Antisense | CTTTCGCTCTGGTCCGTCTT |
| *Wnt5a^(1)^* | Sense | GGAACGAATCCACGCTAAGGGT |
|  | Antisense | AGCACGTCTTGAGGCTACAGGA |
| *Wnt5a-1* | Sense | GTGGCGACTTCCTCTCCG |
|  | Antisense | CGGTCCCCAAAGCCACT |
| *Wnt5a-2* | Sense | ACTTGTTGCTCCGGCCC |
|  | Antisense | CGGTCCCCAAAGCCACT |
| *Wnt5a^(2)^* | Sense | GGCTCCACTTGTTGCTC |
|  | Antisense | TGGCTTAATAACCTCAAACGA |
| *Ctgf* | Sense | CTGCCTACCGACTCGAAGAC |
|  | Antisense | CATTGGTAACTCGGGTGGAG |
| *Cyr61* | Sense | GCTCAGTCAGAAGGCAGACC |
|  | Antisense | GTTCTTGGGGACACAGAGGA |
| *Ankrd1* | Sense | CTGTGAGGCTGAACCGCTAT |
|  | Antisense | TCTCCTTGAGGCTGTCGAAT |
| *Pparg2* | Sense | CTGATGCACTGCCTATGAGCA |
|  | Antisense | ATGCGAGTGGTCTTCCATCAC |
| *IL-6* | Sense | ACAACCACGGCCTTCCCTACTT |
|  | Antisense | CACGATTTCCCAGAGAACATGTG |
| *PAI1* | Sense | GCCTCCTCATCCTGCCTAA |
|  | Antisense | GCCAGGGTTGCACTAAACAT |
| *Fam107a* | Sense | GCTCATCAAACCCAAGAAGCTGC |
|  | Antisense | AGCTCAGGCTTGCTGTCCATAC |
| *FABP4* | Sense | GATGAAATCACCGCAGACGACA |
|  | Antisense | ATTGTGGTCGACTTTCCATCCC |
| *Leptin* | Sense | GCAGTGCCTATCCAGAAAGTCC |
|  | Antisense | GGAATGAAGTCCAAGCCAGTGAC |
| *F4/80* | Sense | CTTTGGCTATGGGCTTCCAGTC |
|  | Antisense | GCAAGGAGGACAGAGTTTATCGTG |
| *TNFa* | Sense | CTGAACTTCGGGGTGATCGG |
|  | Antisense | GGCTTGTCAGTCGAATTTTGAGA |
| *MCP1* | Sense | CCTGCTGTTCACAGTTGC |
|  | Antisense | ATTGGGATCATCTTGCTG |

^(1)^ Primers for *Wnt5a* mRNA of wild type C57BL/6J mice and 3T3-L1 ^(2)^ Primers for *Wnt5a* mRNA of Floxed and FKO mice.

**Supplementary Table S2. Primers used in ChIP-qPCR analyses and genotyping analyses**.

| **PCR Region** | **Strand** | **Sequence** |
| --- | --- | --- |
| *Wnt5a* –0.3kb | Sense | GGCTAGCTGTCCATCAACCG |
|  | Antisense | GCGCCAAATTTTGTCCTTTC |
| *Wnt5a TSS* | Sense | CTGACAATCAGGAGGCGTGA |
|  | Antisense | TACTCAACTGTGGCCCGAGG |
| *Ctgf* –0.1kb | Sense | CTTCTTGGTGTTGTGCTGGA |
|  | Antisense | GATTGATCCTGACCCCTTGA |
| *Wnt5a Exon 2^(1)^* | Sense | GGC TCC ACT TGT TGC |
|  | Antisense | TGG CTT AAT AAC CTC AAA CGA |

^(1)^ primers for genotying Floxed and FKO mice.
